# GH Deficiency and Replacement Therapy in Hypopituitarism: Insight Into the Relationships With Other Hypothalamic-Pituitary Axes

**DOI:** 10.3389/fendo.2021.678778

**Published:** 2021-10-19

**Authors:** Eriselda Profka, Giulia Rodari, Federico Giacchetti, Claudia Giavoli

**Affiliations:** ^1^ Endocrinology Unit, Fondazione Istituto di Ricovero e Cura a Carattere Scientifico (IRCCS) Ca’ Granda, Ospedale Maggiore Policlinico, Milan, Italy; ^2^ Department of Clinical Sciences and Community Health, University of Milan, Milan, Italy

**Keywords:** growth hormone deficiency, hypopituitarism, central hypoadrenalism, central hypothyroidism, hypogonadotropic hypogonadism

## Abstract

GH deficiency (GHD) in adult patients is a complex condition, mainly due to organic lesion of hypothalamic-pituitary region and often associated with multiple pituitary hormone deficiencies (MPHD). The relationships between the GH/IGF-I system and other hypothalamic-pituitary axes are complicated and not yet fully clarified. Many reports have shown a bidirectional interplay both at a central and at a peripheral level. Signs and symptoms of other pituitary deficiencies often overlap and confuse with those due to GH deficiency. Furthermore, a condition of untreated GHD may mask concomitant pituitary deficiencies, mainly central hypothyroidism and hypoadrenalism. In this setting, the diagnosis could be delayed and possible only after recombinant human Growth Hormone (rhGH) replacement. Since inappropriate replacement of other pituitary hormones may exacerbate many manifestations of GHD, a correct diagnosis is crucial. This paper will focus on the main studies aimed to clarify the effects of GHD and rhGH replacement on other pituitary axes. Elucidating the possible contexts in which GHD may develop and examining the proposed mechanisms at the basis of interactions between the GH/IGF-I system and other axes, we will focus on the importance of a correct diagnosis to avoid possible pitfalls.

## Introduction

Growth hormone (GH) deficiency in adults (AGHD) is a complex condition characterized by a well-defined clinical phenotype including modified body composition (increased fat mass and loss of lean muscle mass), intermediate metabolism changes, reduced bone mass, compromised aerobic exercise capacity, impaired quality of life and increased cardiovascular risk profile (1–3).

Response to recombinant human growth hormone (rhGH) replacement therapy has a high inter-individual variability and, though several placebo-controlled and observational studies have provided information on its efficacy and safety, the results are still inconclusive, especially regarding quality of life (QoL) improvement and GH specific mortality reduction (2–5).

In adults, growth hormone deficiency (GHD) is often accompanied by other multiple pituitary hormone deficiencies (MPHD), mainly secondary to organic causes (pituitary tumour mass, surgery or radiation, traumatic brain injury, subarachnoid haemorrhage, hypophysitis, Sheehan’s syndrome, vascular damage, empty sella, hypothalamic infiltrative/inflammatory diseases or pituitary metastasis). Nonetheless, sometimes adult-onset GHD can be idiopathic, due to an impaired somatotroph function in the absence of an underlying pituitary lesion or defect. In this setting, the diagnosis can be extremely challenging due to its subtle manifestations and only an extended use of dynamic GH test may reveal such condition (6, 7).

Less frequently, adult GHD is of childhood origin, reconfirmed at adult height and after the transitional age. Childhood-onset GHD (CO GHD) is mostly occurring as an idiopathic isolated hormone deficiency, being additional MPHD rarely encountered (8, 9). However, there are cases of CO GHD reconfirmed in adulthood and the association with other MPHD represents an important predictive factor of persistent GHD, especially in the presence of organic lesions (i.e. craniopharyngiomas). Indeed, severe GHD tends to reconfirm in more than 90% of organic CO GHD and around 50% of idiopathic GHD (10). Moreover, among CO GHD associated with MPHD, it is worth mentioning congenital aetiologies due to mutations of the transcription factors involved in the embryologic development of the pituitary, namely PROP1, POU1F1 (PIT-1), HESX1, LHX3, LHX4 or SOX2 (11).

Clinical manifestations of MPHD are insidious and strictly dependent on the degree and severity of hormone deficiencies, the gender, the age of onset and the underlying comorbidities. In case organic cause, signs and symptoms related to mass effect can also be present.

The challenging management of MPHD is due to the complex and multifaceted interplay between the GH-IGF-I and other pituitary hormones axis, in which specific signs and symptoms of GHD often coincide with those of other deficits. Moreover, a condition of untreated GHD may mask other underlying pituitary deficiencies, mainly central hypothyroidism (CHT) and hypoadrenalism (CHA). In this setting, an appropriate diagnosis can be possible only after rhGH replacement. On the other hand, the concomitant reduction of other pituitary hormones can alter GH secretion and response to pharmacological stimuli, thus an appropriate replacement therapy is required in order to avoid GHD diagnosis pitfalls (1).

The impact of these interactions is more than theoretical: for instance, since rhGH start may increase cortisol metabolism in patients with MPHD, it is possible that GH treatment initiation could lead to acute adrenal insufficiency by “unmasking” a condition of unsubstituted CHA or require an adjustment of glucocorticoid replacement dosages.

Moreover, several androgens enhance GH effects in peripheral tissues (12) explaining why men are more responsive than young women to rhGH therapy and supporting a sexual dimorphism of rhGH effects at different end-points of the treatment (13).

As aforementioned, the clinical manifestations of AGHD may also be related to other underlying pituitary deficiencies or suboptimal replacement therapies. Thus, a correct diagnosis of hypopituitarism and the subsequent indication of appropriate replacement therapy can be crucial in the detection of the beneficial effects of GH therapy.

**Figure 1:**
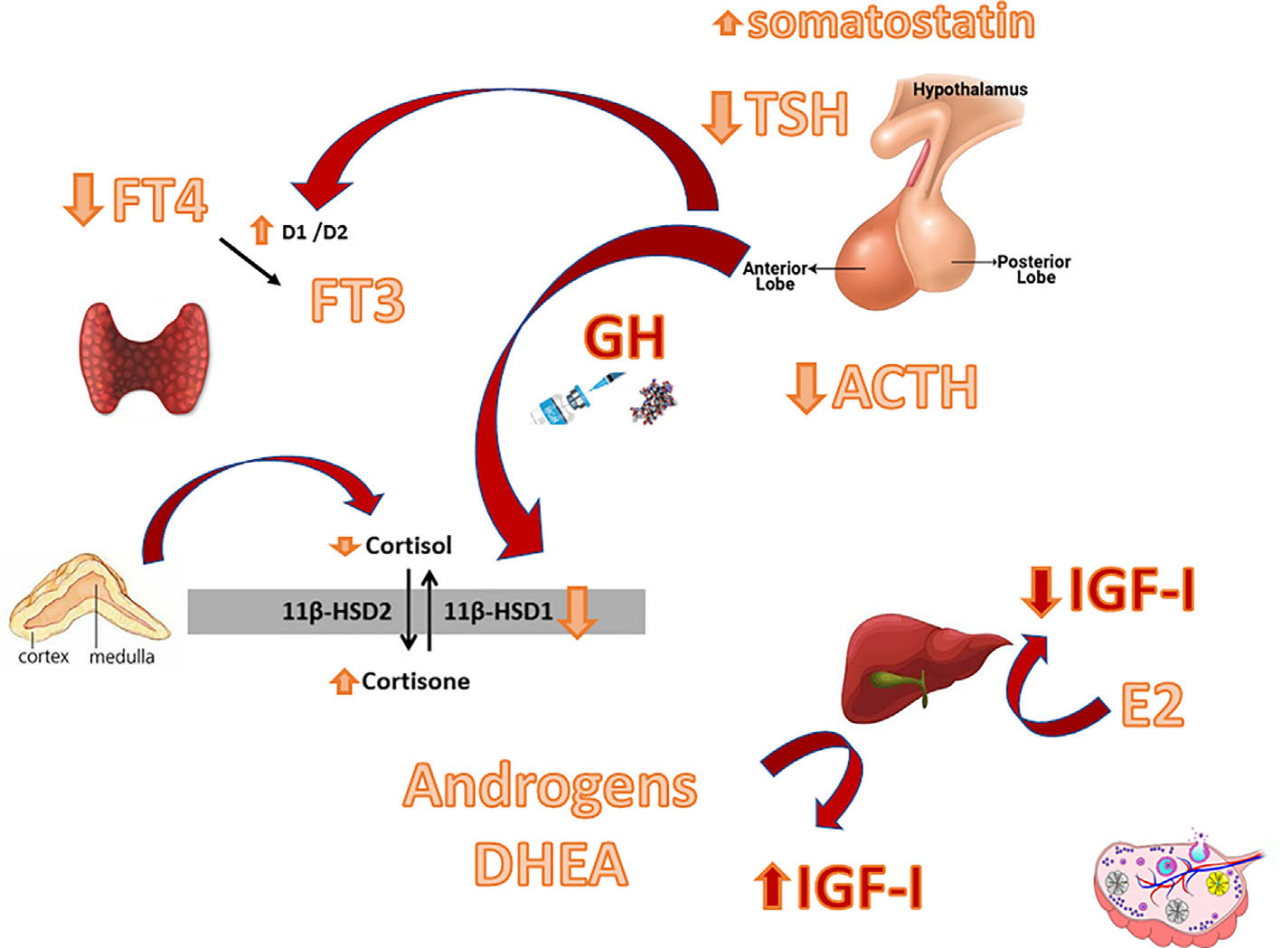
Main interactions between the GH-IGF-I system and other hypothalamic-pituitary axes. D1/D2: Deiodinase type 1/ Deiodinase type 2. 11ßHSD: 11ß-hydroxysteroid dehydrogenase.

By elucidating the possible context in which GHD may develop and by examining the proposed mechanisms and the basis by which the GH/IGF-I system and other axes interact (**Figure 1**), we will enlighten the importance of reaching a correct diagnosis and establishing a correct management to avoid possible pitfalls.

## GH-IGF-I Axis and Hypothalamic-Pituitary-Adrenal Axis

Growth hormone and IGF-I, together with androgens, represent the main anabolic hormones and cortisol the main catabolic one, thus their actions are evidently linked. Several studies have reported a complex relationship between the GH/IGF-I and the hypothalamic-pituitary-adrenal (HPA) axis, both at a central and a peripheral level (14, 15).

At the hypothalamic-pituitary level, altered cortisol and ACTH secretion may affect GH release. Indeed, a substantial body of literature has described that a condition of eucortisolism is required to elicit a GH response to pharmacological testing (16). The clinical importance of this phenomenon is particularly evident in infants with severe ACTH deficiency, when, even in the presence of mutations of transcription factors not involved in GH axis regulation, a severe GHD can resolve with cortisol replacement (17).

At the peripheral level, some studies reported a possible direct effect of GH therapy on cortisol-binding globulin (CBG) levels, but data are contradictory (15, 18–20).

Moreover, the GH-IGF-I axis interplay can act at a tissue level by modulating the activity of 11beta-hydroxysteroid dehydrogenase (11ßHSD): the well-known cortisol-cortisone shuttle (21). The type 1 isoenzyme (11ßHSD1) can be found in the liver, lung, adipose tissue, gonads, pituitary and central nervous system. It is a low affinity NADP(H)-dependent bi-directional enzyme which interconverts inactive cortisone to active cortisol (22). Conversely, Type 2 isoenzyme (11ßHSD2) is a unidirectional, NAD-dependant dehydrogenase, localized in the kidney, placenta, colon and in the salivary glands and has a dehydrogenase activity which converts active cortisol to cortisone (23). In this context, GH modulates cortisol metabolism mainly by inhibiting 11ßHSD1, thus leading to a reduced cortisone activation into cortisol (18, 24–28). The exact mechanism of modulation is far from being clear: *in vitro* studies indicate a dose-dependent inhibition of 11ßHSD1 activity induced by IGF-I, but not by GH (26). Whatsoever, the result is that in the lack of GH an increased amount of cortisol is locally generated. Indeed, it has been hypothesized that some of the phenotypic features of GHD can be explained by an alteration in 11ßHSD1 activity, especially in the liver and in the adipose tissue. In particular, the increased local 11ßHSD1 activity in adipose tissues (24, 29, 30), resulting in increased local cortisol exposure (29), could promote insulin resistance and visceral adiposity which tend to reduce after GH replacement (30, 31), possibly explaining its beneficial effects (32). An intriguing observation has been recently made by Agha and colleagues: the authors reported that 11ßHSD1 activity is regulated differently in patients with different aetiologies of hypopituitarism. In particular, they found that patients with a craniopharyngioma had higher 11ßHSD1 activity even during GH therapy compared to a matched group of patients with NFPAs/prolactinomas, with amplified cortisol production in adipose tissues and liver. The clinical significance of this observation remains unclear but the authors hypothesized that this condition may increase the risk of adverse metabolic outcomes (33). Indeed, regarding metabolic outcomes, patients with craniopharyngiomas seem to have a lower response to GH therapy than those with NFPA (34).

Our group investigated the effect of rhGH on the HPA axis in both adults and children with GHD. The former study was carried-out in 12 patients with adult-onset GHD due to surgically treated pituitary tumours and preserved HPA function, before and during rhGH therapy.

Urinary free cortisol, as well as basal and stimulated serum cortisol levels, were lower on therapy than before and a condition of CHA was unmasked in the majority of subjects. Since no change in CBG was found, the results were mainly ascribed to restoration of 11ßHSD1 activity inhibition induced by GH replacement (27). Nonetheless, in the setting of hypopituitarism, CBG levels need to be considered in women taking oral oestrogen replacement therapy since oestrogens increase CBG and consequently total cortisol levels, but not the unbound active fraction.

The above reported data suggest that in patients with organic hypopituitarism, GH deficiency may mask the presence of CHA. To confirm this observation, the study conducted in 10 children with idiopathic isolated GHD and normal pituitary MRI, showed no changes in the HPA axis during rhGH (35).

The major studies so far available on this topic are reported in **Table 1**.

**Table 1:** Modifications of hypothalamic-pituitary- adrenal axis during GH therapy in AGHD.

Study	N	ACTH-def before rhGH therapy	ACTH-def after rhGH therapy	CBG	MCP	UFC	CoM	F/E
Weaver et al. (18)	19	16	16	↓	NA	↓	↓	↓
Rodriguez-Arnao et al. (19)	14	14	14	↓	↓	NA	NA	NA
Gelding et al. (24)	10	7	7	NA	NA	↔	NA	↓
Tschop et al. (20)	22	16	16	↓	↔	NA	NA	NA
Isidori et al. (15)	30	17	17	↔	NA	NA	NA	NA
Giavoli et al. (27)	12	0	9	↔	↓	↓	NA	NA
Toogood et al. (28)	9	9	9	NA	NA	NA	↔	↓

AGHD, adult growth hormone deficiency; ACTH-def, ACTH deficiency; CBG, cortisol binding globulin; MCP, mean cortisol peak; UFC, urinary free cortisol; CoM, urinary cortisol metabolites; F/E, ratio 11-hydroxy/11-oxo cortisol metabolites; ↔, unchanged; ↓, decreased; NA, not available.

All these observations, taken together, tend to support a strong interplay between the GH/IGF-I and the HPA axis. The clinical impact is particularly evident in patients with MPHD, who may experience a life-threatening adrenal crisis after rhGH initiation in the presence of an untreated CHA.

Thus, in patients with possible MPHD, the integrity of the HPA axis must be evaluated both before GH pharmacological stimulating tests (to avoid diagnostic pitfalls) and after rhGH start. Indeed, an underlying unsubstituted CHA might be unmasked by rhGH therapy, inducing a possible adrenal crisis. Moreover, in patients already under replacement for ACTH deficiency, steroid therapy should be adjusted, especially when using cortisone acetate (1, 36, 37).

## GH-IGF-I Axis and Hypothalamic-Pituitary-Thyroid Axis

It is well ascertained that untreated hypothyroidism is associated with reduced IGF-I and IGF binding protein-3 (IGF-BP-3) and, indeed, even in subclinical hypothyroidism, these reduced levels of IGF-I increase with Levotiroxine (LT4) replacement therapy (38). This phenomenon is easily noticeable in hypothyroid children whose growth failure is reversible by the introduction of LT4 replacement therapy. Moreover, hypothyroidism induces a decrease in GH pulsatility and blunts GH responses to secretory stimuli, changes that are reversible after LT4 introduction, suggesting a possible underlying driven role of thyroid hormones (39).

Therefore, GH provocative tests as well as rhGH replacement therapy should be performed or administered only after the restoration of a condition of euthyroidism. Indeed, as LT4 accelerates cortisol clearance potentially triggering an Addisonian crisis in the presence of an underlying CHA, glucocorticoid replacement therapy should be started first. Thus, in patients with MPHD, hormone replacement therapies must be introduced following a well-defined order: first hydrocortisone, then LT4 (usually after a week), rhGH and, when indicated, sex hormones (36).

However, we have to consider that rhGH therapy can affect the regulation of the HPT axis and thyroid hormone concentrations by several different mechanisms.

Firstly, at a peripheral level, GH induces the extra-thyroidal conversion of T4 to the active hormone triiodothyronine (T3), reducing the concentrations of the inactive form reverse-T3 (rT3) and increasing the T3/T4 ratio (40–45). The effect of GH on circulating T3 levels has been firstly described in animal models (46–48) where GH stimulates T4 conversion to T3. In untreated GHD patients, there is a decreased conversion of T4 to T3, with increased concentrations of rT3 (49). Even if the exact activation pathway under GH control is still unknown, an upregulation of type 2 iodothyroinine deiodinase by GH has been recently described in humans (50). In MPHD, the activity of type 2 deiodinase is usually increased to counterbalance, with a more efficient T3 production, the initial T4 reduction. This compensatory mechanism would be lacking in hypothyroid patients with GHD (50).

Secondly, an interaction at a central level has also been postulated: increased somatostatinergic tone or T3 negative feedback within the thyrotropes, driven by increased T3 production from T4 deiodination, may inhibit TSH release (40–42, 44, 51, 52). In GH deficient adults on rhGH replacement, a significant blunting of the TSH nocturnal surge has indeed been reported (53, 54). However, other studies failed to find TSH variation during rhGH therapy (49). Moreover, whether the interaction between the GH/IGF-I and the HPT axis is directly mediated by GH or through IGF-I is still to be established. Some studies failed to support the influence of IGF-I administration on serum T3 (55). Furthermore, a much higher T3 increase has been described after GH than after IGF-I therapy in GHD patients suggesting that GH has a more direct potent effect on thyroid hormone metabolism (56).

Consistently, in GHD patients under LT4 replacement therapy, rhGH led to a dose-dependent increase in T4 to T3 conversion and a decrease in immunoreactive TSH levels, probably secondary to the increased free T3 in the thyrotropes or to the increased somatostatinergic tone (56, 57). These findings support the crucial role of GH in the HPT homeostasis. Moreover, in hypothyroid patients under LT4 replacement, another possible underlying mechanism is the GH-driven reduction of T4 half-life and the increase of T3 half-life (58) by affecting thyroxine clearance rate or inhibiting LT4 uptake from the gastrointestinal tract (59–61).

When considering GH deficient adults, the first reported results on the interaction with the HPT were controversial, due to the small sample sizes, different study protocols, biochemical analytic methods and criteria for GHD diagnosis, and the use of pituitary GH occasionally contaminated with TSH (45, 62). Nonetheless, subsequent available studies confirmed that, in GHD adults, as in children, rhGH therapy could unmask an underlying CHT. Indeed, a multicenter study evaluated a quite large cohort of patients with either adult or childhood onset severe GHD (17 euthyroid patients and 49 with central hypothyroidism) treated with different rhGH doses (3-12 mg/kg/day) and observed a significant reduction in FT4 and rT3 levels without changes in TSH, FT3 and TBG levels. Interestingly, the fall of FT4 levels was clinically relevant only in patients with organic hypopituitarism (63). A later study confirmed these assumptions in a group of 243 patients, in which the underlying presence of MPHD was found to be the major predictor for CHT development (40). Similar data have been confirmed in long-term follow-up (5 years of rhGH) (64). All in all, the GH-IGF-I and HPT axes interactions have possible tissue-specific effects: indeed, rhGH efficacy on energy expenditure, substrate use and metabolic plasticity can be attenuated by the fluctuations in thyroid hormone levels (65).

**Table 2** summarizes the main studies on this topic.

**Table 2:** Hypothalamic-pituitary-thyroid axis changes during GH replacement therapy in AGHD.

Study	N	CH	TT4/FT4	TSH	TT3/FT3	rT3	% new CH
Jorgensen et al. (45)	21	9	↓/↓	↓NS	↑/↑	↓	0
Amato et al. (62)	9	9	↔/↔	↔	↔/↔	↔	0
Porretti et al. (63)	66	49	NA/↓	↔	NA/↑ transient	↓	47
Agha et al. (39)	243	159	↓NS/↓	↔	↑NS/NA	NA	36
Losa et al. (64)	49	37	NA/↓	↔	NA/↔	NA	17

AGHD, adult growth hormone deficiency; CH, central hypothyroidism, ↔, unchanged; ↓, decreased; ↑increased; NA, not available; NS, not significant.

More uncertainties exist over the effects of GH therapy on thyroid volume (TV) and morphology. Actually, TSH represents the major regulator of both thyroid hormone biosynthesis and thyroid growth. However, IGF-I itself has a proliferative role interacting with its own receptors, largely expressed in thyroid cells (66). Indeed, most acromegalic patients have goiter and IGF-I levels are positively correlated with TV, while hypopituitary patients tend to have reduced TV (67–70).

The finding of unchanged TV during rhGH in TSH- and GH-deficient children, adolescents and adults supports the idea that IGF-I has only a permissive role on the mitogenic action of TSH (71, 72). In fact, an increased TV in patients with congenital isolated GHD was found after 6 months of rhGH therapy (73). Finally, Curtò and collaborators, studying patients with childhood and adult onset GHD before and after 5 years of rhGH therapy, found smaller pretreatment TV in GHD patients than in healthy controls, with increased TV only in patients without concomitant CHT (74).

To summarize, organic GHD can frequently mask a state of CHT, thus it is mandatory to assess and carefully monitor thyroid function before and during rhGH administration, in order to start or adjust LT4 replacement when indicated (1, 37, 75). Indeed, while it is recommended to maintain FT4 in the mid-normal range in patients with CHT, in the presence of a concomitant untreated GH deficiency it would be sensible to aim for higher FT4 levels, given the underlying impairment of T4 to T3 conversion (76, 77). Moreover, most of FT4 variations occur within the first 6 months of therapy, thus the importance of an early revaluation of thyroid function after rhGH initiation (64, 78).

## GH-IGF-I Axis and Hypothalamic-Pituitary-Gonadal Axis

In order to understand the complex interaction between the GH/IGF-I and the hypothalamic-pituitary-gonadal (HPG) axis it is crucial to take into consideration the sexual dimorphism of endogenous GH secretion. Indeed, during the pre-pubertal period, GH and IGF-I levels are similar between boys and girls (79) but in adults spontaneous 24-h GH secretion is approximately two-fold higher in women than in men, mostly due to increased pulse amplitude without a difference in pulse frequency (80). The first gender divergences, indeed, occur during puberty, when pulse GH amplitude in girls tend to precede the one in boys, according to the different timing of the pubertal growth spurt in the two sexes (81). Moreover, GH production declines more quickly with age in women than in men and during menopause this is usually associated with a significant gain in visceral fat mass (82).

Despite this important sexual dimorphism of GH levels, cross-sectional studies have found no difference in serum IGF-I concentrations between women and men (83), though in women a moderate raise of IGF-I levels related to increased GH secretion has been reported in the early follicular and periovulatory phase (82, 84).

The gender-independence of IGF-I levels in healthy adults, despite significantly higher GH concentrations in females, supports the presence of compensated GH resistance in women. This phenomenon is due to a direct inhibitory effect of oestrogen on hepatic but not peripheral IGF-I production. Underlying mechanisms that contribute to this liver sexual dimorphism are pituitary-independent and related to the interaction of oestrogens with their receptors. Namely, the induction of suppressor of cytokine signalling (SOCS)-2 and the inhibition of GHR-Janus kinase (JAK)-2-signal transducer and activator of transcription (STAT)-5 signalling pathway in the liver (85, 86) reduce IGF-I secretion from hepatocytes (87).

Moreover, oral administration of oestrogens introduces an open-loop feedback system during which the continuous and un-physiological suppression of hepatic IGF-I production and release, due to a first pass hepatic effect of oral oestrogen (82), is only partially compensated by increased pituitary GH secretion. In this context, oestrogen replacement discontinuation or omission tends to solve the resistance to GH administration. Serum IGF-I in the GH-deficient state is further lowered by oral oestrogen, but results unaffected by transdermal therapy (88, 89). This phenomenon can explain why IGF-I levels are lower in hypopituitary women than men, despite a similar degree of impaired GH secretion (90). Moreover, women with hypopituitarism tend to be more susceptible to the hepatic effects of oral oestrogens due to the lack of feedback in GH response. Cook et al., indeed, observed that GH requirements in men were not different from those in women not taking oestrogens, but that women taking oral oestrogens required at least a two-fold greater dosage of GH (91).

Interestingly, even in males, many reports have provided robust evidence that oestradiol, rather than testosterone itself, increases GH secretion *via* oestrogen receptor (92, 93) after aromatization from testosterone. In fact, recently, Birzniece and colleagues have shown that the stimulatory effect of testosterone on GH is completely hampered by oestrogen receptor antagonists and by aromatase inhibitors (94).

On the other hand, a study in males revealed that the association of hypogonadotropic hypogonadism (HH) and GHD has an additional lowering effect on testosterone, DHT and oestradiol levels *versus* that seen in isolated HH. This phenomenon supports a synergistic effect of GH/IGF1 on Leydig cell (LC) function (95). In this context, one would have expected an increase in testosterone levels with rhGH therapy. However, the literature available on this topic reported contrasting data. The only studies that showed an increase in testosterone levels included azoospermic (96) or hypogonadal patients (97) with GH and gonadotropin co-treatment. In contrast, in a double-blind placebo controlled trial performed in young males with childhood-onset GHD, Juul et al. (98) concluded that rhGH administration does not influence the HPG axis. Conversely, another study (99) carried out in males with idiopathic isolated GHD, showed that rhGH treatment displays an effect on LC function, increasing testosterone response to chorionic gonadotropin (CG). However, these studies included patients with either idiopathic or organic GHD or varied HPG axis status, being either normogonadic or hypogonadic under treatment with testosterone. Moreover, the high rhGH doses employed in these studies make it difficult to distinguish physiological and pharmacological rhGH effects. In another study on adult males with organic GHD and normal HPG axis we reported a significant decrease in serum testosterone levels strictly related to Sex Hormone Binding Globulin (SHBG) reduction. This suggests the importance of the evaluation of the HPG axis during rhGH treatment, utilizing free calculated testosterone, rather than total testosterone, in order to avoid unnecessary replacement therapy (100).

Moreover, some literature is available on the impact of rhGH treatment on infertility. Males with HH who failed to respond adequately to conventional infertility treatment showed increased testosterone secretion and improved fertility outcomes and sperm production after rhGH adjuvant therapy with gonadotropins (101). In addition, a prospective, open-label, non-randomized observational study of 14 men (26 to 35 years) with normogonadotropic idiopathic oligoasthenospermia found beneficial effects of six months of rhGH treatment on semen volume, count, and motility (102). On the contrary, in a small group of hypogonadotropic hypogonadal azoospermic patients, rhGH replacement therapy for six months, following a previous period of six months of gonadotropin treatment, while increasing testicular volume and testosterone levels, failed to induce the appearance of spermatozoa in the sperm (97). Undoubtedly, the interaction between the GH-IGF-I and the HPG axis plays a role in reproduction and fertility. However, data on the impact of rhGH therapy in non-GHD males are scanty and data on spermatogenesis and fertility in GHD adults, either treated or untreated, are missing.

Similarly, in females, the presence of GH receptors on oocytes suggests a direct action of GH at this level (103). Yet, IGF-I could mediate the reproductive effects of GH, being present in follicular fluid and involved in the cytoplasmic maturation, oocyte capability and granulosa cell function (104). Clinical studies evaluating female patients with suboptimal response to *in vitro* fertilization (IVF), have shown that the co-administration of GH with gonadotropin for controlled ovarian stimulation was associated with a reduction in the gonadotropin requirement, with a higher proportion of successful embryo transfer stage, higher pregnancy and live births rate (105, 106). These outcomes bring to light a possible role for rhGH treatment in oocyte and embryo quality improvement. However, in these patients, endogenous GH secretion was not investigated. When considering GHD, a study by De Boer and Coll reported decreased fertility even in patients without associated hypogonadism (107), suggesting the contribution of GHD to infertility. Giampietro et al. (108) presented four cases of infertility in women with isolated GHD and normal HPG function, in which initiation of rhGH led to efficacious conception and pregnancies. Similarly, in a recent case report by Albu et al, GH therapy contributed to IVF success by improving oocyte competence in a GHD patient. The author concluded that the influence of GH in enhancing oocyte quality should be taken into account in all infertile females with GHD, in order to improve treatment outcome especially when facing previous treatment failure (109). Nevertheless, the responsible mechanisms of GH action on fertility are not fully understood.

Differently from gonadal steroids, in females, DHEA influences the GH/IGF-I axes by increasing IGF-I response thus reducing GH requirement. On the other hand, no rhGH dose adjustment has been necessary in the male group taking testosterone replacement therapy (108, 109). The exact mechanism by which DHEA causes an increase in serum IGF-I levels is still unclear. Some authors have suggested a possible direct stimulatory effect of DHEA on IGF-I hepatic production or an inhibition of IGF-I clearance. On the other hand, DHEA could also enhance GH efficacy acting directly on GH receptors or through testosterone metabolism (110, 111).

To conclude, when treating hypopituitary patients, the gender differences in GH sensitivity and responsiveness are important aspects to take into consideration in clinical practice. In fact, GHD men are more responsive than young women to rhGH therapy, supporting a sexual dimorphism of rhGH effects in different end-points of the treatment. Female patients, indeed, usually require higher rhGH doses to normalize IGF-I levels, especially when receiving oral oestrogen. For this reason, in women with GHD and hypogonadotropic hypogonadism, a transdermal route of oestrogen replacement should be preferred for a cost-effective rhGH treatment.

On the contrary, in males, despite the GH-induced increase in circulating IGF-I by testosterone therapy may suggest the need of lower doses of rhGH, no clinical data have supported a dose reduction during testosterone treatment (112). Moreover, given the above mentioned studies, it is possible to conclude that rhGH treatment does not significantly change the hypothalamic-pituitary-testicular axis metabolism. In this contest, no adjustment of rhGH or testosterone therapy is needed.

Likewise, the reported preliminary data on the influence of the GH/IGF-I axis on fertility does not achieve at present sufficient consensus to be considered in clinical practice.

## Conclusions

In conclusion, the experience developed during the last decades strengthens the view that rhGH replacement therapy is effective and safe in treating GHD in adulthood. Nonetheless, the adult with GHD is a complex patient, in whom the deficit is almost always part of a picture of MPHD. In this context, interactions between replacement therapies have to be taken into account, not only to tailor the best hormonal substitutions, but also to achieve a prompt and accurate diagnosis of hypopituitarism, that is of paramount importance in the management of these patients (**Table 3**). In particular, the state of untreated GHD may mask in a consistent manner a number of cases of central hypoadrenalism and/or hypothyroidism, whose diagnosis becomes possible only after rhGH replacement. Hence, the most recent Guidelines suggest the re-assessment of thyroid and adrenal function during rhGH therapy in patients with organic GHD. Similarly, in patients already under glucocorticoid and LT4 replacement, dosages should be adjusted and usually appropriately increased after rhGH start. In the same context, it is recommended using higher rhGH doses to normalize IGF-I levels in women receiving oral oestrogen and lower doses in women taking DHEA supplement. When possible, a transdermal route of oestrogen replacement should be preferred for a cost-effective rhGH treatment (1, 37).

**Table 3:** GH deficiency and therapy in multiple pituitary hormone deficiency: interactions and dose adjustments.

GH and:	INTERACTION	DOSE ADJUSTEMENT	PITFALL IN DIAGNOSIS
**HPA**	GH suppresses cortisone to cortisol conversion, GH deficiency may result in higher cortisol levels	Re-assess adrenal function through proper dynamic testing after rhGH start	In the presence of ACTH deficiency GH secretion and response to provocative stimuli may be blunted
**HPT**	GH influence T4 to T3 conversion A wide proportion of both euthyroid and hypothyroid patients developed low fT4 levels after rhGH initiation	Increase or start L-T4 after treatment initiation of GH replacement therapy	IGF-I levels are reduced in hypothyroid patients and GH stimulation tests may be blunted
**HPG** **-oestrogens** **-testosterone** **-DHEA**	GH has no clinical influence on gonadal hormone production. -Oestrogens decrease IGF-I hepatic synthesis -Androgens enhance GH effects in peripheral tissues. -DHEA increases the IGF-I levels only in females.	Increase GH dose in women after initiation of oral oestrogen therapy. Decrease GH doses in females after initiation of DHEA therapy. In women with GHD and hypogonadotropic hypogonadism, a transdermal route of oestrogen replacement should be preferred for a cost-effective rhGH treatment.	IGF-I levels are reduced in female patients taking oral oestrogen therapy.

HPA, hypothalamic-pituitary- adrenal axis; HPT, hypothalamic-pituitary-thyroid axis; HPG, hypothalamic-pituitary-gonadal axis.

## Author Contributions

All authors listed have made a substantial, direct and intellectual contribution to the work, and approved it for publication.

## Conflict of Interest

The authors declare that the research was conducted in the absence of any commercial or financial relationships that could be construed as a potential conflict of interest.

The handling editor declared a shared affiliation with the authors at time of review.

## Publisher’s Note

All claims expressed in this article are solely those of the authors and do not necessarily represent those of their affiliated organizations, or those of the publisher, the editors and the reviewers. Any product that may be evaluated in this article, or claim that may be made by its manufacturer, is not guaranteed or endorsed by the publisher.
